# A Case Report on Advanced Hip Tuberculosis: Outcomes of Combined Surgical and Rehabilitative Intervention

**DOI:** 10.7759/cureus.68309

**Published:** 2024-08-31

**Authors:** Gurjeet Kaur, Nikita Gangwani, H V Sharath

**Affiliations:** 1 Physical Medicine and Rehabilitation, Musculoskeletal Physiotherapy, Ravi Nair Physiotherapy College, Datta Meghe Institute of Higher Education and Research (Deemed to be University), Wardha, IND; 2 Pediatric Physiotherapy, Ravi Nair Physiotherapy College, Datta Meghe Institute of Higher Education and Research (Deemed to be University), Wardha, IND

**Keywords:** functional outcomes, multidisciplinary approach, musculoskeletal tb, rehabilitation, total hip replacement (thr), hip tuberculosis

## Abstract

Although rare, musculoskeletal involvement of tuberculosis (TB) sustains this disease as a global health problem. Hip TB presents some unique challenges to its diagnosis and cure because of its specific anatomical and biomechanical properties. Herein, we would like to highlight an integrated approach in the surgical intervention and rehabilitation towards the management of an advanced symptom-bearing 25-year-old female hip TB patient. She had taken treatment for tuberculosis, but even then, her right hip was painful, and movements were severely restricted. Imaging revealed severe destruction of the hip joint; a bone biopsy confirmed tuberculous osteomyelitis of the hip joint. Total hip replacement (THR) revealed the severe destruction of the hip joint by imaging and was found positive by bone biopsy for tuberculous osteomyelitis. The rehabilitation after the surgery consisted of measures for pain control, mobility training exercises, muscle strengthening, and balance training exercises. After six weeks of THR, the patient showed considerable improvement in pain level, flexibility, muscle strength, and functional status during assessments. What is highlighted is the complexity that lies in the management of TB of the hip, which requires the multidisciplinary approach that the case above calls for. In the future, more sophisticated diagnostics and newer therapies should be patient-reported and outcome-oriented. Larger multicenter studies directed to the various populations would be beneficial in this direction. The small size of the study, its single-center dimension, and the short follow-up limited broader applicability and long-term insights.

## Introduction

For thousands of years, tuberculosis (TB), a long-term infectious ailment caused by Mycobacterium tuberculosis, has been mankind’s scourge and still affects millions globally. The disease is rampant in overcrowded, unhygienic, and malnourished areas, creating a persistent factor in many third-world countries. TB primarily manifests as a respiratory disorder but may also occur as extrapulmonary forms, with musculoskeletal pathology accounting for its minute fraction [1]. Musculoskeletal TB represents only 1-3% of all tuberculosis infections. However, India has the highest burden of TB, with approximately one-fourth of the global cases; thus, there are numerous patients with musculoskeletal complications [2]. Hip TB accounts for between 15-20% of all musculoskeletal TB cases, making it clinically challenging for doctors due to its subtle onset and mimicking other musculoskeletal diseases that make diagnosis difficult. After spinal column involvement at 15-20%, the hip is the second most common site affected by osteoarticular TB among bone and joint tuberculosis sites [3]. Infection usually follows a primary lung infection. As such, chondral destructions and bone erosions are different stages through which the tubercular hip progresses before ultimately leading to pain and restricted movement, which are common symptoms of severe arthritis [4]. Delayed diagnosis often results in many patients presenting with an advanced stage of hip tuberculosis. X-ray images may not reflect early-stage hip tuberculosis, which makes it difficult to diagnose this disease [5]. Traditional diagnostic approaches that rely on clinical and radiological evaluations have been surpassed by modern diagnostic methods [6]. Lung infection that is primary to the spreading leads to secondary involvement of the hip joint, resulting in granuloma formation and inflammation that characterize TB in the hip. Cartilage erosion occurs due to infections leading to bone damage, resulting in osteomyelitis and caseous necrosis, which are characteristic features of TB infections [7]. Inflammatory processes over time cause fibrosis and ankylosis, with significant arthritis resulting from immobility [8]. The socioeconomic consequences of TB hip tend to be enormous, particularly in areas where there are many cases [9]. This illness overburdens healthcare systems, negatively affects patients’ quality of life significantly, and causes substantial economic costs associated with prolonged treatment and disability. Reducing the incidence of tuberculosis among the population requires vaccination, public health measures such as Bacillus Calmette-Guérin (BCG) vaccination, efforts aimed at reducing TB transmission in the general community, general improvements in nutrition status, as well as overall better public health. It can be diagnosed through imaging techniques that reveal tissue biopsy for confirmation, but therapeutic intervention involves reducing the incidence of TB. Diagnosis involves imaging and biopsy, with treatment requiring anti-TB medications and possibly surgery [10]. However, advanced hip TB can be destructive to joints, hence making the management of the disease technically difficult, even though this is not a rule. In addition to pre- and postoperative anti-tubercular therapy (ATT), advanced tubercular hip treatment involves surgical interventions such as resection arthroplasty, arthrodesis, and total hip replacement (THR) [11]. THR offers a pain-free, stable joint and restores a normal gait. However, optimal timing of surgery, choice of prosthesis, perioperative ATT, risk of disease reactivation, complications, and long-term durability of the reconstruction are critical factors [12]. Following THR, patients undergo physical rehabilitation to recover from their normal daily activities [13]. Patients receive substantial rehabilitation benefits, including alleviation in pain, enhanced range of motion, improved bearing loads and balance control, increased muscle strength around the hip area, as well a fast walking pace [14]. The paper aims to give an inclusive overview of challenges and management strategies concerning hip TB by emphasizing the role played by physiotherapy in improving patient outcomes following THR. We will explore the complex interplay between medical, surgical, and rehabilitative interventions, highlighting the importance of a multidisciplinary approach in optimizing functional recovery and improving the quality of life for individuals affected by this debilitating condition.

## Case presentation

Patient’s information

This 25-year-old female patient was admitted to the hospital with an inability to walk and unbearable pain in the right hip, which had increased in intensity over the past month. She gives a history of sharp, intermittent pain in the hip that began eight years ago. At that time, she was diagnosed with tuberculosis and treated with four months of anti-tubercular therapy before the medications were stopped. On admission, her X-ray and magnetic resonance imaging scans showed deformities in the acetabulum and femoral capital epiphysis, with diffuse arthritic changes. A biopsy of the femoral head confirmed tuberculous osteomyelitis, thus chronic bone infection due to tuberculosis.

Clinical presentation

Before the evaluation, verbal consent was obtained from the patient. She appeared alert, cooperative, and aware of place, time, and individuals. Her body type was endomorph, with a body mass index (BMI) of 30.43 kg/m². Her vital signs remained constant. This assessment was conducted prior to her surgery. The patient was evaluated in a supine position. On inspection, there was swelling seen around her hip joint; however, there was no discoloration seen. On palpation, grade 3 tenderness was elicited on the anterior and lateral aspects of the hip joint. Pain was assessed using a numeric pain rating scale (NPRS), which revealed a score of 8 out of 10 on movement. Her range of motion was measured using a goniometer, and her strength was tested using a manual muscle test. Both assessments showed pronounced reductions in range of motion and strength secondary to pain and limitation in movement. Table 1 describes the timeline of events.

**Table 1:** Timeline of events TB: tuberculosis; THR: total hip replacement; MRI: magnetic resonance imaging

Timeline	Events	Details
9 Years ago	Initial TB diagnosis	Patient was diagnosed with tuberculosis and underwent four months of anti-tubercular therapy before discontinuing treatment.
8 Years ago	Onset of intermittent hip pain	Patient began experiencing intermittent sharp pain in the right hip.
1 Month ago	Intensification of hip pain	Patient's right hip pain intensified, leading to severe pain and inability to walk.
Admission	Hospital admission	Patient was admitted to the hospital due to inability to walk and severe pain in her right hip.
Diagnostic process	Diagnostic imaging and biopsy	X-ray and MRI scan revealed deformities in the acetabulum and femoral capital epiphysis, with significant arthritic changes. A biopsy confirmed tuberculous osteomyelitis.
Surgery	THR	A total hip replacement was performed on the right hip using aseptic techniques and spinal-epidural anesthesia. Postoperative imaging confirmed successful THR.

On investigation

**Figure 1:**
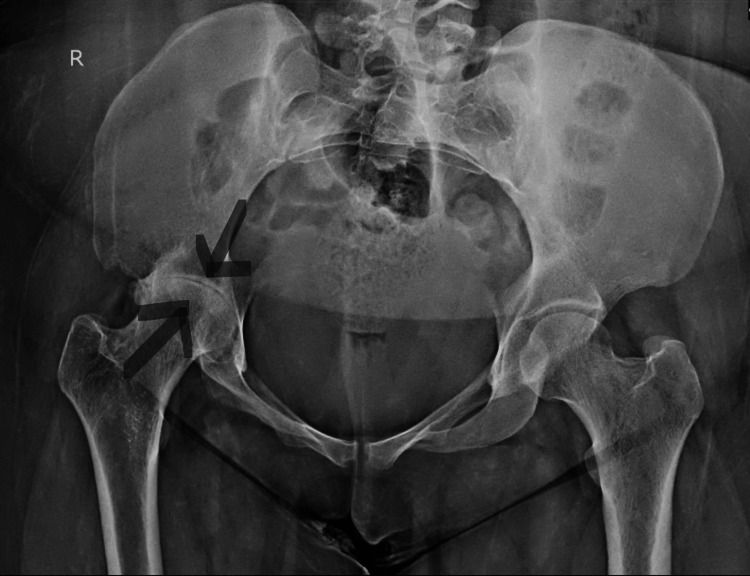
Preoperative X-ray Anteroposterior X-ray of the pelvis and hips indicates significant degenerative changes in the right hip joint, consistent with advanced osteoarthritis or chronic tuberculosis-related osteomyelitis. Findings include marked joint space narrowing, femoral head deformity, acetabular irregularity, sclerosis, erosion, and osteophyte formation.

Before surgery, the patient underwent various tests to evaluate her health and knee injuries, including a complete blood count (CBC), kidney function test (KFT), and liver function test (LFT), all of which were normal, indicating no systemic issues. An X-ray showed significant abnormalities in the right hip joint, including joint space narrowing and deformities of the femoral head and acetabulum. An MRI further revealed these abnormalities and reactive edema in the acetabulum. These pre-surgery evaluations, including CBC, KFT, LFT, and X-ray, are crucial for comprehensive preparation and accurate diagnosis to ensure optimal surgical outcomes. Figure 1 shows a preoperative X-ray.

Surgery details

**Figure 2:**
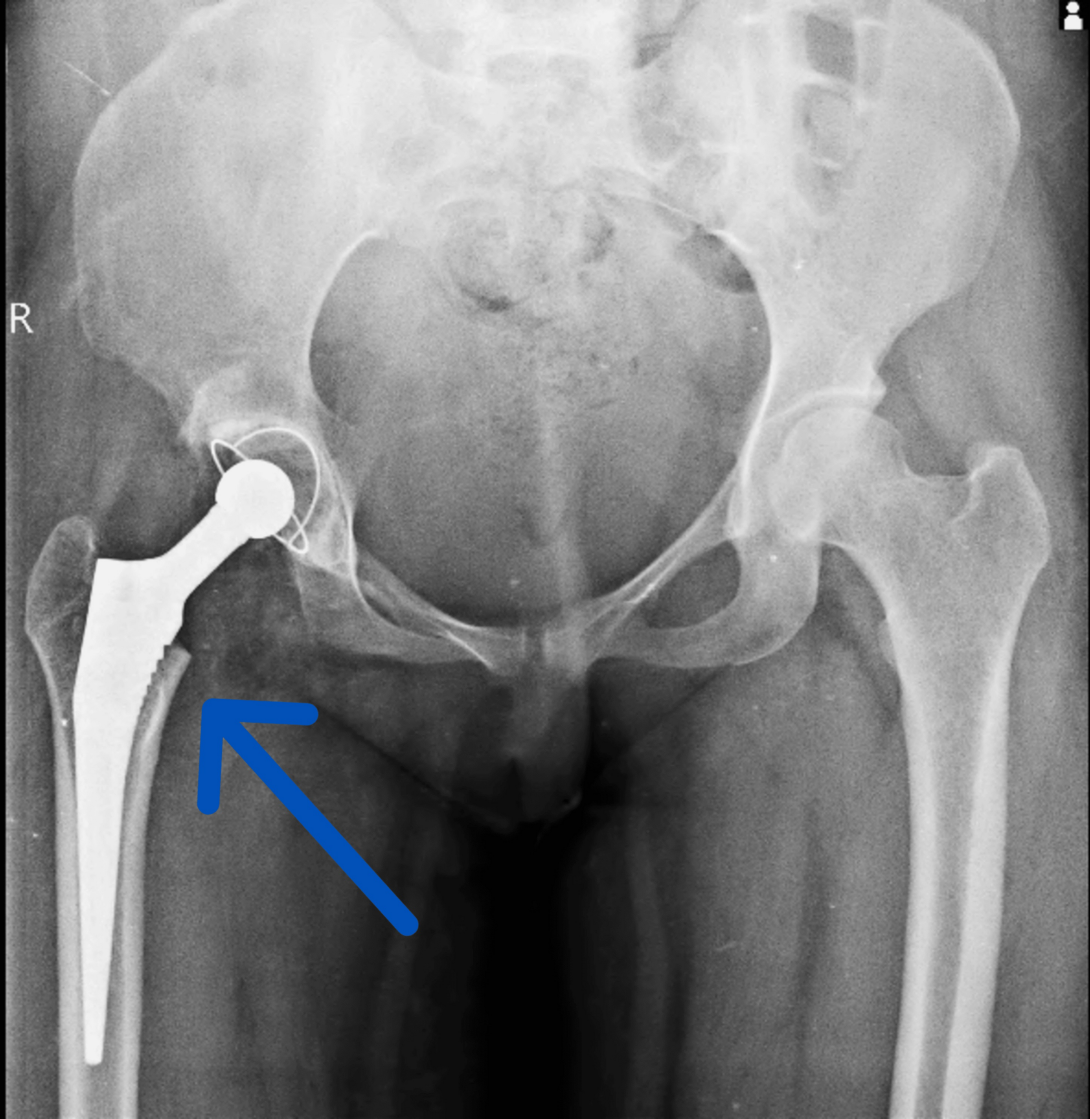
Postoperative X-ray The X-ray shows a cement-less total hip replacement prosthesis.

A THR on the right hip was performed using aseptic techniques and spinal-epidural anesthesia. After a posterior incision and T-shaped capsulotomy, the femoral head was removed, and a press-fit acetabular component was implanted. The femoral canal was prepared, and a cemented femoral stem and head were inserted. The hip joint was relocated, stability confirmed, and the incision was closed with sutures. The patient was then transferred to the recovery unit for monitoring. Figure 2 shows a postoperative image of the hip.

Diagnostic assessment

On postoperative day (POD), the patient was assessed lying on her back with her lower limb abducted and a pillow between her legs for comfort. Pain levels decreased from 8/10 to 6/10 by POD three. Range of motion and muscle strength were evaluated to determine improvements post-surgery. Functional outcome measures, including the Harris hip score, lower extremity functional scale (LEFS), and hip disability and osteoarthritis outcome score (HOOS), were used to assess the patient's daily function and recovery. All assessments occurred on POD three, coinciding with the start of physiotherapy, with follow-up evaluations conducted at six weeks to monitor progress. The range of motion (active) is presented in Table 2.

**Table 2:** Range of motion (ROM) Measurements of active range of motion for the right hip and knee were recorded before surgery, on postoperative day three before the start of rehabilitation, and again at the six-week mark following rehabilitation.

Joint and movements (right side)	Preoperative	Postoperative day 3/Pre-rehabilitation day 1	Post-rehabilitation (week 6)
Hip joint			
Flexion	0-10°	0-20°	0-60°
Extension	0-5°	0-10°	0-20°
Abduction	0-20°	0-30°	0-40°
Adduction	0-10º	N/A	0-30°
Internal rotation	0-5°	0-10°	0-20°
External rotation	0-5°	0-10°	0-20°
Knee joint			
Flexion	0-40°	0-20°	0-70°
Extension	40°-0	20°-0	70°-0

Manual muscle testing (MMT) of the muscles around hip joint and knee joint are graded with modified medical research council (MMRC) presented in Table 3.

**Table 3:** Manual muscle testing Grade 0: No muscle contraction detected; Grade 1: Slight trace of muscle contraction; Grade 2-: Partial range of motion with gravity eliminated; Grade 2: Complete range of motion with gravity eliminated; Grade 2+: Less than half the range of motion against gravity; Grade 3-: More than half the range of motion against gravity, but not complete; Grade 3: Full range of motion against gravity; Grade 3+: Full range of motion against gravity with minimal resistance; Grade 4-: Full range of motion against gravity with less than moderate resistance; Grade 4: Full range of motion against gravity with moderate resistance; Grade 4+: Full range of motion against gravity with nearly maximum resistance; Grade 5: Normal strength (full range of motion against gravity with maximum resistance)

Joint and movements (right side)	Preoperative	Postoperative day 3/Pre-rehabilitation day 1	Post-rehabilitation (week 6)
Hip joint			
Flexion	2/5	3/5	4/5
Extension	2/5	3/5	3+/5
Abduction	2/5	3/5	3+/5
Adduction	2/5	2/5	3/5
Internal rotation	2/5	2/5	3/5
External rotation	2/5	2/5	3+/5
Knee joint			
Flexion	2/5	3/5	3+/5
Extension	2/5	3/5	3+/5

Physiotherapy protocol

Recovery from a hip replacement occurs in phases. The first week emphasizes safe bed movement and using walking aids while managing swelling. Weeks one to three involve increased mobility, pain management, and strength and balance exercises. Finally, weeks three to six focus on building endurance through walking and stationary bikes, along with advanced balance exercises to encourage independent living, all while taking precautions to protect the new hip [15].

Phase 1

From day three to one week post-surgery, the goals are to educate the patient on dislocation precautions, reduce inflammation, swelling, and pain, and ensure independence in bed mobility and transfers to a chair or toilet. Gait training with appropriate devices and a home exercise program will also be initiated to support recovery and improve function. Table 4 depicts Phase 1 of the rehabilitation.

**Table 4:** Phase 1 rehabilitation protocol ROM: range of motion

Intervention	Frequency	Intensity	Time	Type	Rationale
Isometric quadriceps, hamstring, and glute exercises	5 times per week	Moderate (perform to fatigue but not exhaustion)	3 sets of 10 repetitions	Isometric exercises	Gentle, targeted exercises strengthen muscles, stabilize joints, and reduce pain, accelerating recovery.
Active assisted/active ROM exercises	5 times per week	Moderate (perform to fatigue but not exhaustion)	3 sets of 10 repetitions each exercise	Active assisted exercise	Straight leg raises and knee slides restore knee strength and flexibility, aiding recovery.
Bed mobility and transfer training	Daily	Light to moderate (based on patient’s capacity)	As needed for practice and skill acquisition	Bed mobility and transfer training exercises	Boost independence, prevent falls, and accelerate recovery through improved strength and coordination for safe mobility.
Gait training on flat surfaces with walker	Daily	Light to moderate (based on patient’s capacity)	1-2 rounds or as tolerated by the patient	Gait training on flat surfaces using a walker	Helps patients regain mobility and confidence in their movement.
Progress to stair training with support as needed	As tolerated (2-3 times per week)	Moderate (based on patient’s capacity)	1 flight or as tolerated by the patient	Stair training with support	Enhances lower body strength and confidence for safe stair climbing, promoting independence.

Phase 2

In Phase 2 (week one to week three post-surgery), the goals are to enhance range of motion while preventing hip dislocation, reduce inflammation and swelling, and strengthen hip girdle muscles for stability. Proprioceptive training will improve body awareness and joint positioning, and endurance training will boost overall cardiovascular fitness. Table 5 depicts Phase 2 of rehabilitation.

**Table 5:** Phase 2 rehabilitation protocol ROM: range of motion

Intervention	Frequency	Intensity	Time	Type	Rationale
Active assisted/assisted stretching for hip abduction ROM	Daily	As tolerated by the patient	3 sets of 10-15 reps	Stretching exercise	Enhances flexibility, reduces pain, and improves functional mobility.
Isometric quadriceps, hamstring, and gluteal exercises	Daily	Hold each contraction for 5-10 seconds	10-15 reps, 2-3 sets	Isometric exercise	Builds muscle strength and stability without joint movement, aiding in rehabilitation and preventing injury.
Heel slides	Daily	Moderate, as tolerated	10-15 reps, 2-3 sets	Range of motion exercise	Enhances knee joint flexibility and range of motion while gently strengthening the quadriceps and hamstrings.
Gait training	Daily	Gradual reduction of assistive device use	Throughout the day	Gait training exercises	Promotes independence and strengthens muscles, enhancing balance and coordination while restoring natural walking patterns.
Balance/proprioception training: Weight-shifting activities	Daily	Moderate	10-15 reps, 2-3 sets	Balance exercises	Improves stability, coordination, and joint awareness, enhancing functional movement and reducing fall risk.
Closed kinetic chain activities	Daily	Moderate	10-15 reps, 2-3 sets	Closed kinetic chain balance exercises	Enhances neuromuscular control and overall stability.
4-way straight leg raise	Daily	Moderate	10-15 reps each direction, 2-3 sets	Straight leg raise exercises in four directions	Strengthens key hip muscles, enhancing stability, balance, and overall lower extremity strength.
Sit-to-stand	Daily	Moderate	10-15 reps, 2-3 sets	Functional mobility exercises	Improves functional mobility and daily living independence.
Stationary bike	Start at 3rd week, daily	Progress resistance as tolerated	10-20 minutes	Cardiovascular and lower extremity strength exercises using a stationary bike	Supports cardiovascular fitness and lower extremity strength during rehabilitation.

Phase 3

In Phase 3 (week three to week six post-surgery), the goals are to progressively increase range of motion for near-normal hip function, reduce inflammation and manage pain, and advance strengthening exercises for muscle tone and joint stability. Proprioception and balance exercises will be enhanced, endurance training will improve fitness and stamina, and efforts will focus on achieving independence in daily activities and mobility without assistance. Table 6 describes Phase 3 of rehabilitation.

**Table 6:** Phase 3 rehabilitation protocol

Intervention	Frequency	Intensity	Time	Type	Rationale
Assess hip, knee, and trunk stability	-	-	-	-	-
Open chain activities	3-4 times per week	Moderate	2-3 sets of 10-15 reps	Dynamic and static exercises	Enhances joint stability and control through isolated movements.
Initiate endurance program	-	-	-	-	-
Walking	Daily	Moderate pace	20-30 minutes	Low-impact cardiovascular exercise	Builds cardiovascular endurance and supports overall mobility.
Stationary bicycle	3-4 times per week	Progress resistance as tolerated.	20-30 minutes	Low-impact cardiovascular exercise	Enhances cardiovascular fitness and lower extremity strength.
Initiate and progress age-appropriate balance exercises	-	-	-	-	-
Static balance exercises	Daily	Moderate	2-3 sets of 10-15 reps	Static balance exercises	Improves postural control and stability.
Dynamic balance exercises	Daily	Moderate	2-3 sets of 10-15 reps	Dynamic balance exercises	Enhances dynamic stability and functional balance.
Single-leg stance variations	Daily	Moderate	2-3 sets of 10-15 reps per leg	Single-leg stance exercises	Strengthens lower extremity muscles and improves unilateral balance.
Balance board or unstable surface training	3-4 times per week	Moderate to challenging	10-15 minutes	Balance board or unstable surface exercises	Enhances proprioception, coordination, and balance under challenging conditions.

Post-surgery precautions include avoiding provocative positions such as hip flexion, adduction, and internal rotation and restricting hip flexion to no more than 90 degrees. The patient should not cross their legs or feet and should avoid rolling or lying on the unoperated side for the first six weeks. Twisting the upper body when standing is to be refrained from, and sleeping on the back is recommended for the initial six weeks. The use of a shower chair or elevated seat at home is advised, and bathing in a flexed and bent-down position in the tub should be avoided for eight to 12 weeks. Aids should be used to assist in putting on underwear, socks, and shoes to prevent deep hip flexion angles for six weeks.

Results

Table 7 likely presents a comparison of outcome measures like pain, range of motion, and functional scores before surgery and after rehabilitation. It would highlight improvements or changes in these metrics, supported by statistical analysis to determine significance. The overall goal is to assess the effectiveness of the rehabilitation program in patient recovery.

**Table 7:** Outcome measure LEFS: lower extremity functional score; HOOS: hip disability and osteoarthritis outcome score

Outcome measure	Preoperative	Post-rehabilitation
Harris hip score	20.04%	43.35%
LEFS	2/80	17/80
HOOS	9.4%	48%

The table demonstrates significant improvements in various outcome measures before and after rehabilitation. The Harris hip score, assessing hip joint function, increased from 20.04% preoperatively to 43.35% post-rehabilitation, indicating substantial enhancement in hip function. LEFS improved from 2/80 to 17/80, reflecting enhanced lower limb functional abilities. Additionally, the HOOS rose from 9.4% to 48%, showing a marked reduction in symptoms and improved quality of life. Overall, these results highlight the positive impact of rehabilitation on hip function and lower extremity performance.

## Discussion

This case report highlights a thorough management approach for a 25-year-old female patient with advanced hip TB, emphasizing the crucial role of integrating surgical intervention with rehabilitation. The patient had a history of tuberculosis and intermittent hip pain that evolved into severe pain and disability, requiring a detailed diagnostic process including X-rays, MRI, and biopsy, which confirmed tuberculous osteomyelitis. Accurate diagnosis and treatment planning in such cases depend on thorough imaging and histopathological analysis. The surgical treatment involved a meticulously planned THR, highlighting the need for precision and careful handling of tissues to minimize complications. Post-surgery, a structured rehabilitation protocol was essential for the patient’s recovery. The initial phase focused on pain management and early mobilization with gentle range of motion exercises and gradual weight-bearing to prevent joint stiffness and support tissue healing [16]. Rehabilitation was divided into three phases. Phase 1 (day three to week one) included isometric exercises and basic mobility training. In Phase 2 (week one to week three), the focus shifted to active-assisted exercises, balance training, and reducing dependence on assistive devices. Phase 3 (week three to week six) emphasized improving endurance, stability, and proprioception through dynamic activities and progressive resistance exercises. This structured progression was critical in ensuring a safe and gradual return to normal function while minimizing complications and re-injury risks. Supporting literature reinforces this approach. Research by Matheis and Stöggl demonstrated that targeted mobilization and strength training of hip muscles starting on the third day post-THR significantly improves hip range of motion and gait performance within the first week [17]. Additionally, Han et al.'s studies showed that a six-week rehabilitation program could effectively restore hip joint function, and monitored home exercise programs are comparably effective as standard rehabilitation practices for early recovery after total knee replacement [18]. Patient progress was assessed using various outcome measures, including the Harris hip score, LEFS, and HOOS. Preoperative scores were notably low, reflecting the severe impact of hip tuberculosis on the patient’s functionality and quality of life. Post-rehabilitation, significant improvements in all outcome measures were observed, confirming the effectiveness of the combined surgical and rehabilitation interventions.

This is despite the fact that hip tuberculosis management is still challenging, especially as it is usually diagnosed late and requires prolonged treatment [19]. Socioeconomics, this is especially true for areas where there is a high prevalence of TB cases; it puts a strain on healthcare systems, placing a physical toll on the patient, apart from the economic toll. The main preventive measures to lower the incidence of TB are avoiding contracting the illness, BCG vaccination, good public health infrastructure, and addressing malnutrition [20]. Future studies should embrace larger samples, if not multicenter studies, for better generalizability and further extended follow-up to allow for the assessment of long-term outcomes. All these are important in fine-tuning the management strategies in an attempt to improve the quality of life for patients with hip tuberculosis.

## Conclusions

This case report describes a 25-year-old female with tuberculous hip osteomyelitis who underwent successful total hip replacement and structured rehabilitation with significant functional improvement. It therefore places a premium on an early diagnosis, appropriate surgical intervention, and comprehensive rehabilitation as cornerstones of the management of tuberculosis of the hip for optimal patient outcomes.
